# Outcomes of venovenous-extracorporeal membrane oxygenation bridging in lung transplant recipients with panel reactive antibody positivity

**DOI:** 10.1007/s10047-025-01539-2

**Published:** 2025-11-13

**Authors:** Austin Chang, Yudai Miyashita, Benjamin Louis Thomae, Amanda Kamar, Taisuke Kaiho, Chitaru Kurihara

**Affiliations:** 1 Division of Thoracic Surgery, Department of Surgery, Northwestern University Feinberg School of Medicine, 676 N. St Clair St, Suite 650, Chicago, IL 60611 USA; 2 Division of Pulmonary and Critical Care, Department of Medicine, Northwestern University Feinberg School of Medicine, 676 N. St Clair St, Suite 650, Chicago, IL 60611 USA

**Keywords:** Lung transplantation, Extracorporeal membrane oxygenation, Acute respiratory distress syndrome, Panel reactive antibody positivity

## Abstract

**Background:**

Lung transplantation improves survival in advanced lung disease, but calculated Panel Reactive Antibody (cPRA) positive patients are at higher risk for adverse outcomes. It is unclear if sensitization poses additional risks in patients bridged to transplantation with venovenous extracorporeal membrane oxygenation (VV-ECMO). This study compares outcomes between cPRA-positive and -negative patients bridged with VV-ECMO.

**Methods:**

Among 411 transplant recipients from an institutional lung transplantation database (January 2018–March 2025), 52 patients were bridged with VV-ECMO. Of these, 14 were cPRA-positive and 38 were cPRA-negative. Univariate and multivariate analyses evaluated early post-transplant complications and survival outcomes in cPRA-positive and -negative groups.

**Results:**

Rates of post-transplant complications such as primary graft dysfunction (PGD) in any grade (85.7% cPRA-positive vs. 76.4% cPRA-negative, *p* = 0.48), PGD grade 3 (35.7% vs. 50.0%, *p* = 0.53), acute kidney injury (AKI) (64.3% vs. 68.4%, *p* = 1.00), dialysis requirement (21.4% vs. 34.2%, *p* = 0.51), deep vein thrombosis (DVT) (71.4% vs. 55.3%, *p* = 0.35), and pulmonary embolism (PE) (7.1% vs. 18.4%, *p* = 0.42) were similar. 1-year survival rate (85.1% vs. 82.9%) and overall survival (*p* = 0.81) were not significantly different.

**Conclusions:**

Post-transplant outcomes and survival were similar between cPRA-positive and -negative groups, suggesting that PRA status in VV-ECMO bridged lung transplant recipients is not associated with worse outcomes. VV-ECMO may remain a viable bridge to lung transplantation, even in sensitized patients.

**Supplementary Information:**

The online version contains supplementary material available at 10.1007/s10047-025-01539-2.

## Background

Lung transplantation is a critical intervention for patients with end-stage lung diseases, offering a potential for extended survival and improved quality of life [1, 2]. Despite advancements in surgical techniques and postoperative care, lung transplantation remains complex, with various challenges affecting patient outcomes. One significant challenge is the management of patients who deteriorate while on the waiting list for a transplant. In such cases, venovenous extracorporeal membrane oxygenation (VV-ECMO) has emerged as a pivotal bridging therapy [3–6]. VV-ECMO has been increasingly utilized as a bridge to lung transplantation, with studies demonstrating its potential to stabilize critically ill patients and improve their candidacy for transplant [7–9].

While the benefits of VV-ECMO in bridging to lung transplantation are established, it is not without its own risks. VV-ECMO is associated with post-transplant complications including bleeding and thromboembolic events, dialysis requirement, and bloodstream infection [10, 11]. Additionally, VV-ECMO may contribute to sensitization—the development of anti-HLA (human leukocyte antigen) antibodies—due to blood transfusions and prolonged extracorporeal circulation, though this relationship is not fully understood [12–14]. Sensitization remains a critical issue in transplant immunology. Sensitized patients face a higher risk of graft rejection and complications post-transplant, leading to poorer outcomes and increased mortality [15].

Understanding the impact of VV ECMO bridging in high-risk patients is crucial, as it may influence transplant eligibility and long-term patient survival. Given the independent risks associated with both VV-ECMO and sensitization, it is important to assess whether the combination of the two further compromises outcomes or if VV-ECMO remains a viable bridging strategy, even in sensitized patients. However, current literature lacks comprehensive retrospective analyses that focus on outcomes of VV-ECMO bridging in sensitized patients, specifically. We hypothesize that cPRA-positive patients bridged to lung transplantation with VV-ECMO experience non-inferior early post-transplant outcomes and survival compared with cPRA-negative patients and the primary objective of this study is to determine if sensitization—defined by cPRA status—poses additional risks in patients bridged to lung transplantation with VV-ECMO. This study compares early post-transplant complications and survival outcomes between cPRA-positive and -negative patients bridged with VV-ECMO.

## Methods

### Study design

The study was approved by the Institutional Review Board of Northwestern University (STU00207250 and STU00213616). The need for patient consent for data collection was waived by the institutional review board due to the retrospective nature of this study.

Patient data were collected retrospectively using electronic medical records and stored in a database at the Northwestern University Medical Center in Chicago, Illinois, USA. Adult patients who underwent lung transplantation with preoperative VV-ECMO as bridge to lung transplant at our institution between January 2018 and March 2025 were included. Multiorgan transplant recipients and re-transplant recipients were excluded from the study. Data on patient demographics, comorbidities, donor characteristics, preoperative laboratory values, intraoperative and postoperative outcomes, and panel reactive antibodies (PRA) results were collected. All blood test results, including panel reactive antibody (PRA) levels, were obtained after the initiation of ECMO support and immediately before transplantation.

PRA positivity was determined using calculated PRA (cPRA) with anti-HLA antibody typing. Patients with cPRA 30% were considered cPRA-positive and highly sensitized, in keeping with the threshold proposed by Tinckam et al. for peri-operative desensitization in lung transplantation [16]. The remaining patients were in the cPRA-negative group. Development of de novo donor specific antibodies (DSA) was defined as any new DSA titers after lung transplantation.

### ECMO indication criteria

Prior to lung transplantation, all intubated patients were treated by a multidisciplinary team in accordance with the guidelines of the National Heart, Lung, and Blood Institute’s ARDS Network [17]. Indications for ECMO evaluation included refractory hypoxemia with PaO_2_ less than 55 mmHg, pulse oximetry oxygen saturation less than 88%, and pH level less than 7.2. Patients were evaluated with lung-protective mechanical ventilation with a plateau pressure of less than 35 mmHg, neuromuscular blockade, and prone positioning, according to recommendations from the Extracorporeal Life Support Organization [18]. Central venous catheters (CVCs) were replaced every seven days, even if there were no signs of infection. New CVC replacements were performed in the same vein and not in a wire-based replacement, but in a different area or new vein. Weekly surveillance cultures for all ECMO patients were used to monitor bloodstream infections simultaneously, consistent with our previous study [19].

### Anticoagulation during VV-ECMO support

Patients did not receive continuous anticoagulation unless there was a specific indication, such as deep venous thrombosis (DVT) or pulmonary embolism (PE), and there was no monitoring of bleeding parameters, such as activated clotting time or activated partial thromboplastin time, which is consistent with our previous study (19). All patients who were not receiving continuous systemic anticoagulation received 5,000 U of subcutaneous unfractionated heparin every 8 h as a prophylactic dose to prevent DVT. VV-ECMO flow was maintained at a minimum of 3.0–3.5 L/min, consistent with our recent reports, to reduce thrombotic complications in the ECMO circuit [20].

### Peri-operative management for sensitized patients

Patients were treated with peri-operative plasmapheresis and eculizumab if cPRA 30% or positive crossmatch. Patients with cPRA < 30% and negative crossmatch did not receive plasmapheresis with eculizumab (Table S1). Once a donor offer was accepted for each sensitized patient, the initial session of plasmapheresis with eculizumab was coordinated. The eculizumab dosing regimen and timeline was modified from treatment of heart transplant recipients [21]. The initial dose of 1200 mg was given pre-operatively following the initial session of plasmapheresis and continued at 900 mg daily for the first four post-operative days. Weekly dosing of 900 mg then resumed on POD 10 for an additional two weeks and was escalated to 1200 mg at weeks six and eight. The initial doses of basiliximab and methylprednisolone were administered intra-operatively. Methylprednisolone, anti-thymocyte globulin (ATG), and intravenous immunoglobulin (IVIG) were dosed based on ideal body weight. ATG was dosed at 1 mg/kg. IVIG was dosed at 300 mg/kg with a final dose of 1 g/kg. Post-operatively, all patients were maintained on a triple immunosuppressive regimen including a calcineurin inhibitor, mycophenolic acid, and corticosteroid. All patients were ensured to be vaccinated for meningococcus prior to therapy and were started on penicillin prophylaxis for at least three months following completion of the protocol [22].

### Definition of complication

#### Primary graft dysfunction (PGD)

PGD was defined based on the ISHLT guideline [23], and graded by PaO2/FiO2 ratio as follows; Grade 1: PaO2/FiO2 ratio > 300; Grade 2: PaO2/FiO2 ratio is 200–300; Grade 3: PaO2/FiO2 ratio < 200. The use of ECMO for bilateral pulmonary edema on chest X-ray was classified grade 3.

#### Acute kidney injury (AKI)

AKI was defined using the Risk, Failure, Loss of kidney function, and End-stage kidney disease classification [24].

### Statistical analysis

Continuous data are shown as median ± standard deviation and discrete data are shown as number (%). The Mann–Whitney U test was used to compare independent continuous variables. Fisher’s exact test was used to compare categorical variables. The Kaplan–Meier method was used to estimate survival, and the log-rank test was performed to compare survival between the groups. Odds ratios (OR) were obtained using a univariate and multivariate logistic regression analysis. Hazard ratios (HR) were obtained using univariate Cox proportional hazards analysis; variables with *p* < 0.05 on univariate Cox analysis were subsequently included in multivariate Cox models. Statistical significance was set at *p* < 0.05. All statistical analyses were performed using the R version 4.5.1. In an exploratory analysis we repeated all outcome comparisons in the full transplant cohort (n = 411) to place the VV-ECMO findings in broader context (Supplemental Tables S1–S4, Figure S1).

## Results

### Patient demographics

A total of 411 lung transplant recipients were included in this analysis, of whom 52 required VV-ECMO (12.7%) as a bridge to transplantation (Table 1). During the study period, all extracorporeal support for transplant candidates was VV-ECMO; no VA or VAV configurations were employed. Of these 52 patients bridged with VV-ECMO, 14 were cPRA-positive (26.9%) and 38 were cPRA-negative (73.1%). Composite cPRA ranged from 0 to 100% with a median of 4.0% (IQR 0.0–40.0%) (Supplemental Fig. S2). The dashed line in Fig. S1 marks the 30% threshold used to define the cPRA-positive group; no natural inflection above this point was observed. Patients in the cPRA-positive group had significantly more females (78.6% vs. 36.8%, *p* = 0.01). There were no significant differences in age or etiology of end-stage lung disease. The groups had similar prevalence of smoking history, hypertension, diabetes, chronic kidney disease, bilateral lung transplant, and pre-operative blood transfusions. cPRA-positive patients had significantly higher sodium and albumin levels (145.0 [139.8–147.5] vs. 140.0 [138.8–142.3] mEq/L, *p* = 0.01; 4.0 [3.6–4.3] vs. 3.4 [3.1–3.9] g/dl, *p* = 0.001). Other pre-transplant laboratory values and donor characteristics were similar between the groups.

**Table 1:** Characteristics of patients

Variable	VV-ECMO bridge		*p* value
	cPRA negative (n = 38)	cPRA positive (n = 14)	
Recipient factors			
Age, years	49.5 (34.8–58.0)	53.0 (38.3–57.0)	0.51
Female	14 (36.8%)	11 (78.6%)	0.01
BMI, kg/m2	25.2 (21.6–27.6)	28.0 (25.3–31.1)	0.03
BSA, m2	1.9 (1.7–2.0)	1.9 (1.7–2.1)	0.85
Smoking history	11 (29.0%)	1 (7.1%)	0.14
Hypertension	16 (42.1%)	8 (57.1%)	0.37
Diabetes	10 (26.3%)	3 (21.4%)	1.00
CKD	2 (5.3%)	0 (0.0%)	1.00
Bilateral	37 (97.4%)	14 (100.0%)	1.00
Pre-op RBC transfusion			
Number within 4 weeks	34 (89.4%)	10 (71.4%)	0.19
Unit within 4 weeks	2.0 (1.0–7.0)	1.5 (0.0–6.5)	0.49
Number within 1 week	19 (67.9%)	9 (64.3%)	1.00
Unit within 1 week	1.0 (0.0–2.0)	1.0 (0.0–2.5)	0.91
Etiology			0.05
ILD	12 (31.6%)	3 (21.4%)	
PAH	2 (5.3%)	1 (7.1%)	
ARDS	22 (57.9%)	9 (64.3%)	
Other	2 (5.3%)	1 (7.1%)	
Laboratory			
Hemoglobin, g/dL	8.2 (7.3–8.9)	7.5 (7.1–8.3)	0.24
WBC, 1,000/mm3	9.9 (7.7–13.7)	9.7 (8.1–15.3)	0.73
Platelets, 1,000/mm3	140.0 (101.8–206.0)	160.5 (142.8–223.0)	0.15
Sodium, mEq/L	140.0 (138.8–142.3)	145.0 (139.8–147.5)	0.01
BUN, mg/dL	16.0 (13.0–25.3)	23.0 (14.3–42.3)	0.09
Creatinine, mg/dL	0.6 (0.4–0.8)	0.6 (0.4–0.8)	0.69
ALT, U/L	19.0 (11.0–30.0)	18.0 (12.5–42.3)	0.61
AST, U/L	23.0 (17.5–36.0)	22.0 (21.0–45.5)	0.63
Albumin, g/dL	3.4 (3.1–3.9)	4.0 (3.6–4.3)	0.001
Total bilirubin, mg/dL	0.9 (0.5–1.5)	0.8 (0.6–1.1)	0.39
INR	1.2 (1.1–1.2)	1.2 (1.1–1.3)	0.68
Donor			
Age, years	36.5 (25.0–43.3)	34.5 (25.8–44.3)	0.89
Female	18 (47.4%)	5 (35.7%)	0.54
Cause of death			0.007
Anoxia	13 (34.2%)	7 (50.0%)	
Head trauma	16 (42.1%)	2 (14.3%)	
Stroke	8 (21.1%)	4 (28.6%)	
Other	1 (2.6%)	1 (7.1%)	

Continuous data are shown as median (range) and discrete data are shown as number (%). BMI, body mass index; BSA, body surface area; CKD, chronic kidney disease; PRA, panel reactive antibody; ILD; interstitial lung disease; COPD, chronic obstructive pulmonary disease; PAH, pulmonary arterial hypertension; ARDS, Acute Respiratory Distress Syndrome; WBC, white blood cell; BUN, blood urea nitrogen; AST, aspartate aminotransferase; ALT, Alanine aminotransferase; INR, international normalized ratio. *Unknown cases were excluded

### Intraoperative and postoperative outcomes

Intraoperative parameters were similar between groups: median operative time was 8.2 h (5.4–9.5) in cPRA-negative versus 7.6 h (6.8–10.2) in cPRA-positive patients (*p* = 0.70); median pRBC transfusion 7.0 units (5.0–10.3) versus 11.0 units (6.8–16.3; *p* = 0.14); FFP 2.0 units (1.0–6.0) versus 3.5 units (2.0–6.3; *p* = 0.26); platelets 2.0 units (1.0–4.0) in both groups (*p* = 0.76); and ischemic time 5.8 h (5.1–6.6) versus 6.0 h (5.7–6.8; *p* = 0.18) (Table 2).

**Table 2:** Intraoperative outcomes of lung transplant recipients

Variable	VV-ECMO bridge		*p* value
	cPRA negative (n = 38)	cPRA positive (n = 14)	
Intraoperative outcome			
Operative time (hours)	8.2 (5.4–9.5)	7.6 (6.8–10.2)	0.70
Intra-op blood transfusion(unit)			
pRBC	7.0 (5.0–10.3)	11.0 (6.8–16.3)	0.14
FFP	2.0 (1.0–6.0)	3.5 (2.0–6.3)	0.26
Plt	2.0 (1.0–4.0)	2.0 (1.0–4.0)	0.76
Ischemic time (hours)	5.8 (5.1–6.6)	6.0 (5.7–6.8)	0.18
Postoperative outcomes			
de novo DSA	7 (18.4%)	5 (35.7%)	0.27
PGD			
Any grade	28 (76.4%)	12 (85.7%)	0.48
Grade3	19 (50.0%)	5 (35.7%)	0.53
AKI	26 (68.4%)	9 (64.3%)	1.00
Dialysis	13 (34.2%)	3 (21.4%)	0.51
CVA	1 (2.6%)	0 (0.0%)	1.00
Bowel ischemia	1 (2.6%)	0 (0.0%)	1.00
Digital ischemia	4 (10.5%)	1 (7.1%)	1.00
DVT	21 (55.3%)	10 (71.4%)	0.35
PE	7 (18.4%)	1 (7.1%)	0.42
ICU stay (days)*	19.5 (11.8–25.8)	19.5 (11.5–27.8)	0.92
Post transplant ventilator (days)*	4.0 (2.0–15.5)	6.5 (2.0–18.3)	0.69
Hospital stay (days)	37.5 (23.8–48.5)	35.0 (23.5–41.0)	0.46
Follow-up period (days)	632.5 (304.8–1387.5)	787.0 (383.0–1179.5)	0.96
Follow-up period (days)	632.5 (304.8–1387.5)	787.0 (383.0–1179.5)	0.96

Continuous data are shown as median (interquartile range) and discrete data are shown as number (%). pRBC, packed red blood cells; FFP, fresh frozen plasma; Plt, platelets; VA ECMO, veno-arterial extracorporeal membrane oxygenation; DSA, donor specific antibody; PGD, primary graft dysfunction; AKI, acute kidney injury; CVA, cerebrovascular attack; DVT, deep vein thrombosis; PE, pulmonary embolism; ICU, intensive care unit. *Unknown cases were excluded

Postoperative complications did not differ significantly by cPRA status. De novo DSA occurred in 18.4% versus 35.7% (*p* = 0.27). Any-grade PGD was seen in 76.4% versus 85.7% (*p* = 0.48) and PGD grade 3 in 50.0% versus 35.7% (*p* = 0.53). Rates of AKI (68.4% vs 64.3%; *p* = 1.00), dialysis (34.2% vs 21.4%; *p* = 0.51), CVA (2.6% vs 0%; *p* = 1.00), bowel ischemia (2.6% vs 0%; *p* = 1.00), and digital ischemia (10.5% vs 7.1%; *p* = 1.00) were comparable. Thromboembolic events—DVT (55.3% vs 71.4%; *p* = 0.35) and pulmonary embolism (18.4% vs 7.1%; *p* = 0.42)—also showed no significant differences.

**Fig. 1:**
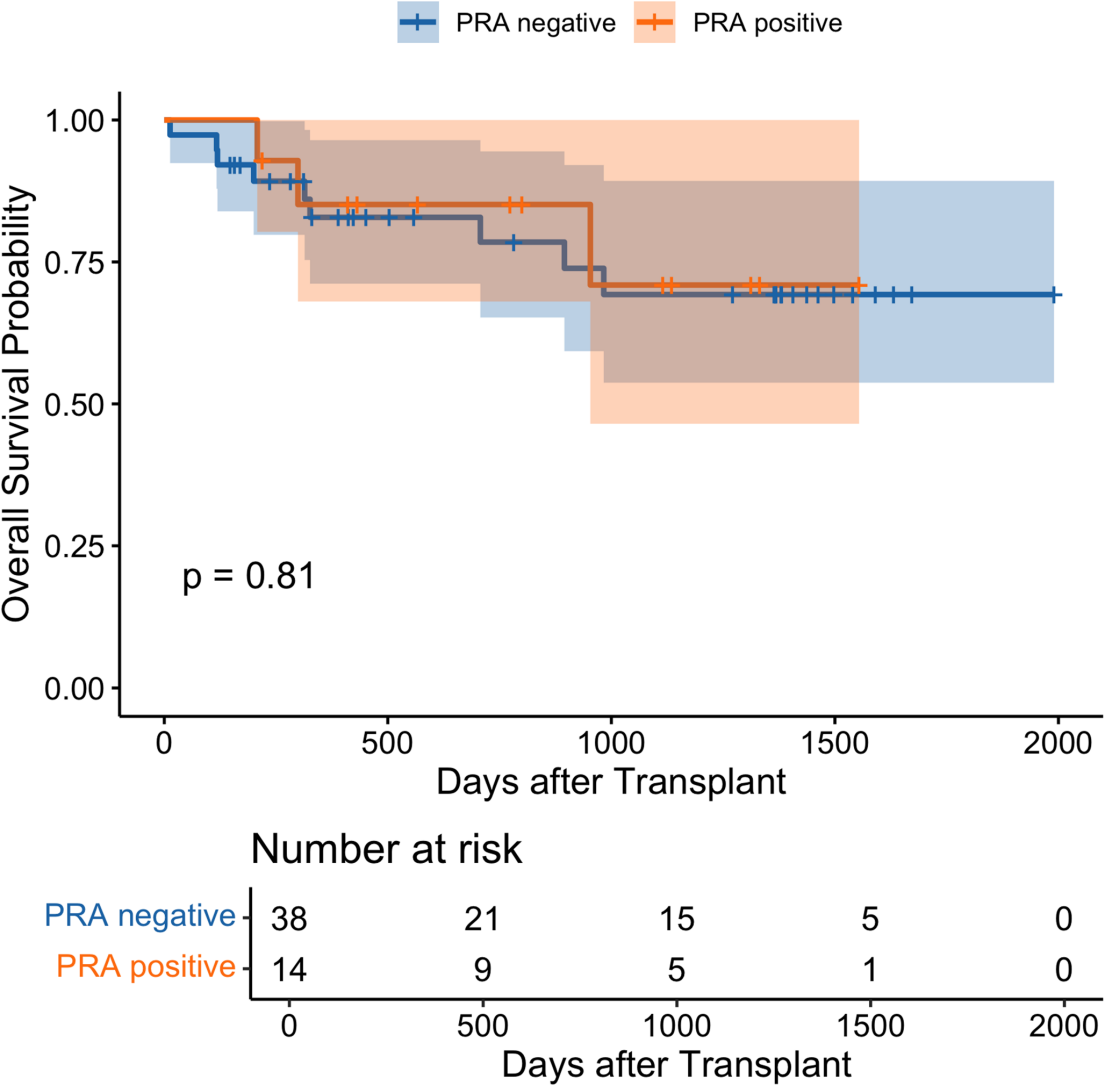
Overall Survival of cPRA-positive and -negative Patients with VV-ECMO Bridge to Lung Transplant. Kaplan–Meier survival curves comparing overall survival between cPRA-positive and -negative patients with VV-ECMO bridge prior to lung transplantation. The red line represents the cPRA-negative group, and the blue line represents the cPRA-positive group

Median ICU stay was 19.5 days (11.8–25.8) in cPRA-negative and 19.5 days (11.5–27.8) in cPRA-positive patients (*p* = 0.92); median ventilator duration 4.0 days (2.0–15.5) versus 6.5 days (2.0–18.3; *p* = 0.69); and median hospital stay 37.5 days (23.8–48.5) versus 35.0 days (23.5–41.0; *p* = 0.46). Follow-up duration did not differ (632.5 d [304.8–1387.5] vs 787.0 d [383.0–1179.5]; *p* = 0.96). Kaplan–Meier survival curves also demonstrated no significant difference in overall survival for VV-ECMO bridged patients with and without cPRA positivity (*p* = 0.81) (Fig. 1). Among all 411 recipients, overall survival did not differ between cPRA-negative and cPRA-positive patients (*p* = 0.85) (Supplemental Fig. S2).

### Predictors of PGD Grade 3

Univariate and multivariate logistic regression analyses were performed to identify factors associated with PGD grade 3 (Table 3). In the univariate analysis, higher creatinine (Odds Ratio (OR) 18.53, [1.47–233.52, 95% confidence interval (CI)], *p* = 0.02) was significantly and lower albumin (OR 0.42 [0.15–1.14, 95% CI], *p* = 0.09) was tend to associated with PGD grade 3. In the multivariate analysis, higher creatinine (OR 23.17 [1.51–356.36, 95% CI], *p* = 0.02) remained independent predictors of PGD grade 3. cPRA positivity was not a significant predictor of PGD grade 3 (OR 0.56 [0.16–1.97, 95% CI], *p* = 0.90).

**Table 3:** Univariate and multivariate logistic regression analysis as a predictor of PGD grade3

Variable	Univariate			Multivariate		
	Odds ratio	95% CI	*p* value	Odds ratio	95% CI	*p* value
Recipient factors						
Age, years	0.96	0.92–1.01	0.09			
Female	1.15	0.39–3.44	0.80			
BMI, kg/m2	0.99	0.87–1.12	0.83			
BSA, m2	0.49	0.04–5.93	0.58			
Smoking history	1.22	0.34–4.45	0.76			
Hypertension	0.98	0.33–2.91	0.97			
Diabetes	1.51	0.43–5.33	0.52			
CKD	1.17	0.07–19.83	0.91			
Pre-op blood transfusion						
Within 4 weeks	1.09	0.96–1.23	0.17			
Within 1 week	1.37	0.94–1.99	0.10			
cPRA > 30%	0.56	0.16–1.97	0.36			
Etiology						
ILD	1.5	0.45–5.01	0.51			
ARDS	0.91	0.30–2.75	0.86			
Laboratory						
Hemoglobin, g/dL	1.25	0.77–2.03	0.37			
WBC, 1,000/mm3	1.06	0.95–1.18	0.29			
Platelets, 1,000/mm3	0.99	0.99–1.00	0.21			
Sodium, mEq/L	0.97	0.86–1.10	0.62			
BUN, mg/dL	1	0.96–1.05	0.85			
Creatinine, mg/dL	18.53	1.47–233.52	0.02	23.17	1.51–356.36	0.02
ALT, U/L	1.01	0.98–1.05	0.35			
AST, U/L	1.01	0.99–1.03	0.18			
Albumin, g/dL	0.42	0.15–1.14	0.09	0.38	0.13–1.15	0.09
Total bilirubin, mg/dL	1.93	0.84–4.42	0.12			
INR	0.28	0.01–10.78	0.49			
Donor						
Age, years	0.97	0.93–1.02	0.26			
Female	0.6	0.20–1.82	0.37			
Intraoperative outcome						
Operative time (hours)	0.86	0.66–1.12	0.26			
Ischemic time (hours)	0.95	0.81–1.12	0.57			

DSA, donor specific antibody; BMI, body mass index; BSA, body surface area; CKD, chronic kidney disease; PRA, Panel Reactive Antibody; WBC, white blood cell; BUN, blood urea nitrogen; AST, aspartate aminotransferase; ALT, Alanine aminotransferase; INR, international normalized ratio. *Unknown cases were excluded

### Predictors of survival

A univariate Cox proportional hazards analysis was performed to identify factors associated with overall survival (Table 4). In the univariate analysis, only pulmonary embolism was significantly associated with mortality (Hazard Ratio (HR) 5.30 [1.67–16.81, 95% CI], *p* = 0.005). cPRA positivity was not a significant predictor of overall survival (HR 0.85 [0.23–3.15, 95% CI], *p* = 0.81). Other recipient factors, pre-transplant laboratory values, intraoperative outcomes, and postoperative outcomes were not associated with mortality.

**Table 4:** Univariate cox proportional hazard analysis as a predictor of survival

Variable	Univariate analysis		
	Hazard ratio	95% CI	*p* value
Recipient factors			
Age, years	1.02	0.97–1.07	0.36
Female	1.54	0.49–4.88	0.46
BMI, kg/m2	1.02	0.89–1.16	0.78
BSA, m2	1.72	0.13–22.47	0.68
Smoking history	2.11	0.63–7.02	0.23
Hypertension	1.05	0.33–3.35	0.94
Diabetes	1.49	0.45–4.98	0.51
CKD	3.05	0.37–24.84	0.30
Bilateral	6.25	0.77–50.84	0.09
PRA	0.85	0.23–3.15	0.81
Etiology			
ILD	0.75	0.16–3.50	0.71
ARDS	2.43	0.52–11.26	0.26
Laboratory			
Hemoglobin, g/dL	0.87	0.49–1.54	0.64
WBC, 1,000/mm3	0.92	0.80–1.07	0.28
Platelets, 1,000/mm3	1.00	0.99–1.00	0.31
Sodium, mEq/L	0.95	0.83–1.08	0.43
BUN, mg/dL	1.00	0.96–1.05	0.98
Creatinine, mg/dL	1.71	0.18–16.57	0.64
ALT, U/L	1.01	0.98–1.04	0.71
AST, U/L	1.01	0.99–1.02	0.30
Albumin, g/dL	0.56	0.22–1.44	0.23
Total bilirubin, mg/dL	1.29	0.86–1.92	0.22
INR	0.12	0.00–6.85	0.31
Donor			
Age, years	1.01	0.97–1.06	0.56
Female	0.95	0.30–2.99	0.93
Intraoperative outcome			
Operative time (hours)	1.15	0.85–1.57	0.37
Ischemic time (hours)	0.77	0.46–1.29	0.32
Postoperative outcomes			
PGD			
Any grade	1.29	0.34–4.82	0.71
Grade3	2.03	0.64–6.41	0.23
AKI	2.24	0.49–10.22	0.30
Dialysis	2.19	0.59–8.14	0.24
DVT	1.71	0.46–6.31	0.42
PE	5.30	1.67–16.81	0.005

BMI, body mass index; BSA, body surface area; CKD, chronic kidney disease; PRA, Panel Reactive Antibody; WBC, white blood cell; BUN, blood urea nitrogen; AST, aspartate aminotransferase; ALT, Alanine aminotransferase; INR, international normalized ratio; DSA, donor specific antibody; PGD, primary graft dysfunction; AKI, acute kidney injury; DVT, deep vein thrombosis; PE, pulmonary embolism; ICU, intensive care unit. *Unknown cases were excluded. *Unknown cases were excluded

## Discussion

VV-ECMO as a bridge to lung transplantation may improve transplant candidacy for patients with end-stage lung disease. However, both VV-ECMO and sensitization are independently associated with worse post-transplant outcomes. This study investigated the early mortality risk and survival in sensitized patients bridged to lung transplant with VV-ECMO. We found that 10.0% of our patients required VV-ECMO as a bridge to lung transplantation, of which 57.5% were cPRA-positive and 42.5% were cPRA-negative. Post-transplant complication rates, one-year survival, and overall survival were comparable between cPRA-positive and -negative groups. Despite the independent risks of VV-ECMO and sensitization, these findings suggest that cPRA positivity does not confer additional risk in lung transplant recipients bridged with VV-ECMO.

Although VV-ECMO as a bridge to lung transplantation has clear benefits for critically ill patients on the waiting list, it carries known risks. Some of the most frequent complications with perioperative ECMO use are the increased incidence of bleeding and thromboembolic events, which are associated with worse survival [25, 26]. However, in our cohort, we found no significant difference in intraoperative blood transfusions, post-transplant DVT, and post-transplant PE between cPRA-positive and -negative patients. These findings suggest that while sensitization itself may predispose to immune-mediated complications, it does not necessarily potentiate the bleeding and thromboembolic risks of VV-ECMO. Furthermore, our anticoagulation and ECMO protocols may have mitigated potential added risks in sensitized patients [20].

Renal injury is another common early complication following lung transplantation. ECMO has been linked to higher rates of AKI and increased need for renal replacement therapy, both of which are associated with greater hospital mortality [27, 28]. Sensitized patients often require more intensive immunosuppression, which can contribute to nephrotoxicity [29]. Additionally, increased intraoperative transfusions from both VV-ECMO and sensitization may exacerbate the risk of renal injury through volume overload [30]. In our study, rates of AKI (69.6% cPRA-positive vs. 76.5% cPRA-negative, *p* = 0.73) and dialysis requirement (34.8% vs. 41.2%, *p* = 0.75) were substantial but not significantly different between groups. These findings suggest that sensitization does not confer additional risk for renal injury in patients bridged to lung transplantation with VV-ECMO, despite the baseline risks associated with VV-ECMO and sensitization. Although VV-ECMO may delay early post-transplant recovery [31], we found that cPRA positivity did not further prolong post-transplant ventilator use, ICU stay, or hospital length of stay. This may be because cPRA-positive patients did not experience significantly higher rates of post-transplant complications such as AKI, DVT, or PE which can hinder recovery.

Sensitization, particularly the development of anti-HLA antibodies, poses a significant risk for graft rejection and poor outcomes in lung transplant recipients [32, 33]. Both sensitization and VV-ECMO have been associated with increased risk for primary graft dysfunction (PGD), a major predictor of early morbidity and mortality after lung transplantation [34, 35]. We found that rates of PGD in any grade (69.6% cPRA-positive vs. 64.7% cPRA-negative, *p* > 0.99) and PGD grade 3 (39.1% vs. 41.2%, *p* > 0.99) were high in both groups, reflecting inherent risks in VV-ECMO and lung transplantation. However, rates of PGD development were not greater the cPRA-positive group, suggesting that the additive impact of sensitization may be minimal or insignificant in the context of VV-ECMO bridging, where baseline PGD risk is already elevated [35]. Finally, we found that 1-year survival rates and overall survival were similar between cPRA-positive and -negative groups. cPRA positivity was not a significant predictor of either PGD grade 3 or overall survival. It is possible that our aggressive perioperative immunosuppressive regimen of plasmapheresis, eculizumab, and ATG employed in cPRA-positive patients may have helped attenuate the immunologic risks associated with sensitization, contributing to similar PGD development rates and survival outcomes. Moreover, recent studies have shown that one-year and overall survival after lung transplantation do not differ between VV-ECMO-bridged and non-bridged patients, suggesting that with appropriate perioperative desensitization and immunosuppression, even sensitized patients bridged on VV-ECMO may achieve outcomes comparable to non-VV-ECMO recipients [36, 37] evertheless, further investigation is needed to clarify the role and efficacy of perioperative immunosuppression strategies in this high-risk population.

This study has several limitations. First, it is a single-center retrospective analysis, which may limit the generalizability of the findings and the power of the study. Given the modest sample size, non-significant *p*-values may reflect insufficient power rather than true equivalence. Observed trends—such as numerically lower PE incidence in cPRA-positive patients—may warrant investigation in larger, multicenter cohorts. Although cPRA-positive and -negative groups did not differ significantly in pre-transplant traits, laboratory values, or donor characteristics, the cPRA-negative cohort was on average three years older, had nearly threefold greater smoking prevalence, and received lungs from donors two years older—imbalances that, despite non-significance in univariate Cox models, may contribute to residual confounding. Although we did not have the power to perform propensity score matching, cPRA-positive and -negative groups did not have significant differences in pre-transplant traits, lab values, or donor characteristics. Additionally, while we adjusted for multiple variables in the multivariate analysis, residual confounding factors may still influence the results. Therefore, studies using matched analyses to control for these confounding variables may be necessary. Third, there is no data available about cPRA before VV-ECMO support. Finally, while cPRA-positive patients had comparable one-year survival outcomes to their cPRA-negative counterparts, the long-term impact of sensitization in VV-ECMO bridged patients beyond one-year post-transplant remains unclear. Our cohort includes only patients who survived to receive lung transplantation; we lack data on VV-ECMO–bridged candidates who died or were weaned prior to transplant. Thus, our findings may underestimate the accurate risk profile of VV-ECMO in sensitized wait-listed patients. Future prospective studies should capture the full spectrum of outcomes—including pre-transplant mortality and weaning—in order to mitigate selection bias. Also, our registry lacks detailed VV-ECMO management metrics—such as support duration, circuit exchange frequency, documented thromboses, and ECMO-related infections—which likely modulate both immunologic sensitization and transfusion exposure. Prospective data collection with standardized ECMO event logging will be necessary to delineate these contributions. We only captured pre-transplant pRBC transfusion within the 4-week and 1-week windows; FFP and platelet exposures over the whole duration of VV-ECMO support were not available. Given that the majority of our cohort had prolonged VV-ECMO runs for ARDS, unmeasured cumulative transfusions may have influenced cPRA levels. Future prospective studies will comprehensively record all blood product exposures during ECMO to better delineate their role in allo-sensitization. Notably, our institution’s protocol includes aggressive perioperative desensitization—plasmapheresis with eculizumab ± ATG/IVIG for cPRA ≥ 30% [38]—and a low-intensity anticoagulation strategy during VV-ECMO (subcutaneous heparin only unless otherwise indicated) [20]. These measures may have attenuated both immunologic and thrombotic complications in our sensitized cohort and therefore may not fully reflect outcomes in centers using different desensitization or anticoagulation regimens. We acknowledge that our modest sample size—particularly in the cPRA-positive VV-ECMO subgroup—limits statistical power. Non-significant differences may represent type II error, and trends warrant evaluation in a larger population. To enhance generalizability, future validation through multicenter prospective registries—such as the Extracorporeal Life Support Organization (ELSO) database or the ISHLT Lung Transplant Registry—is essential. Such collaborative efforts can capture diverse ECMO management practices and larger patient numbers to confirm our findings.

In conclusion, this study demonstrates that sensitized lung transplant recipients bridged with VV-ECMO experience similar intraoperative and post-transplant outcomes—including amounts of blood transfusions, rates of PGD and AKI, and hospital length of stay—compared to their non-sensitized counterparts. Although transplant teams should still carefully evaluate the independent risks associated with VV-ECMO and sensitization, the comparable 1-year and overall survival outcomes indicate that VV-ECMO remains a viable bridge-to-transplant strategy, even in highly sensitized patients.

## Supplementary Information

Below is the link to the electronic supplementary material.

Supplementary Material 1

## Data Availability

The datasets generated and/or analyzed during the current study are available from the corresponding author on reasonable request.
