# Supplementary material for: Outcomes of venovenous-extracorporeal membrane oxygenation bridging in lung transplant recipients with panel reactive antibody positivity
Source: J Artif Organs. 2025 Nov 13;29(1):2. doi: 10.1007/s10047-025-01539-2 (PMC12615553; doi:10.1007/s10047-025-01539-2)
Supplement: Supplementary file 1 — Supplementary Material 1 [file 10047_2025_1539_MOESM1_ESM.pdf]

Supplementary information for

Outcomes of Venovenous-Extracorporeal Membrane Oxygenation Bridging in Lung Transplant Recipients with Panel Reactive Antibody Positivity

Austin Chang, Yudai Miyashita, Benjamin Louis Thomae,

Amanda Kamar, Taisuke Kaiho, Chitaru Kurihara

Corresponding Author:

Chitaru Kurihara, MD

E-mail; chitaru.kurihara@northwestern.edu

This PDF file includes:

Supplemental Figure S1~S2

Supplemental Table S1-S4

Supplemental Figure1

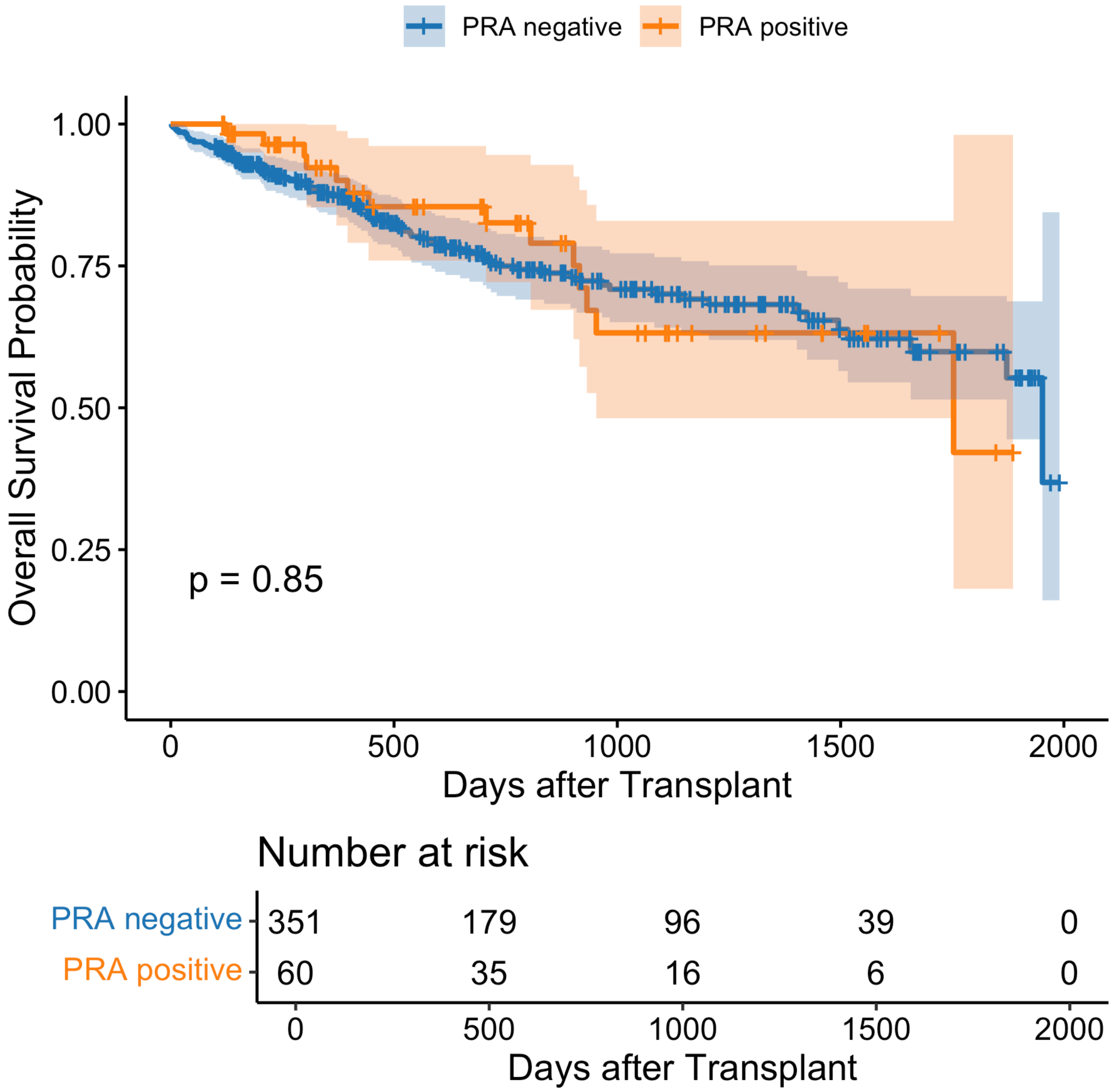

Supplemental Figure2

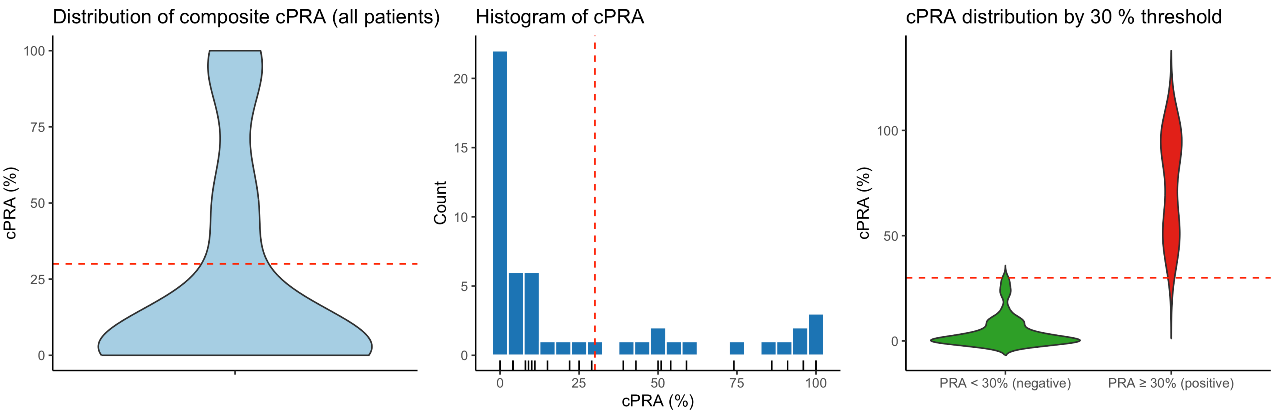

Supplemental Table 1. Characteristics of patients

Variable	No VV-ECMO bridge		VV-ECMO bridge	
	cPRA negative (n=313)	cPRA positive (n=46)	cPRA negative (n=38)	cPRA positive (n=14)
Recipient factors				
Age, years	64 (57—69)	58 (49—67)	49.5 (34.8-58.0)	53.0 (38.3-57.0)
Female	115 (36.7%)	37 (80.4%)	14 (36.8%)	11 (78.6%)
BMI, kg/m2	26.4 (22.1—29.7)	26.7 (23.8—29.3)	25.2 (21.7—27.2)	28.0 (26.1—31.0)
BSA, m2	1.88 (1.70—2.06)	1.79 (1.63—1.87)	1.89 (1.68—2.02)	1.91 (1.69—2.08)
Smoking history	169 (54.0%)	20 (43.5%)	11 (28.9%)	1 (7.1%)
Hypertension	188 (60.1%)	19 (41.3%)	16 (42.1%)	8 (57.1%)
Diabetes	96 (30.7%)	15 (32.6%)	10 (26.3%)	3 (21.4%)
CKD	27 (8.6%)	3 (6.5%)	2 (5.3%)	0 (0.0%)
Bilateral	183 (58.5%)	26 (56.5%)	37 (97.4%)	14 (100.0%)
Pre-op blood transfusion(unit)				
within 4weeks	4 (1.3%)	1 (2.2%)	34 (89.5%)	10 (71.4%)
within 1week	4 (1.3%)	1 (2.2%)	27 (71.1%)	9 (64.3%)
Etiology				
ILD	152 (48.6%)	17 (37.0%)	12 (31.6%)	3 (21.4%)
COPD	71 (22.7%)	7 (15.2%)	0 (0.0%)	0 (0.0%)
PAH	23 (7.3%)	5 (10.9%)	2 (5.3%)	1 (7.1%)
ARDS	13 (4.2%)	4 (8.7%)	22 (57.9%)	9 (64.3%)
other	54 (17.3%)	13 (28.3%)	2 (5.3%)	1 (7.1%)
Laboratory				
Hemoglobin, g/dL*	12.00 (10.80—13.70)	11.20 (10.00—12.50)	8.15 (7.30—8.90)	7.50 (7.10—8.30)
WBC, 1,000/mm3*	8.8 (7.0—11.2)	9.2 (7.4—11.6)	9.9 (7.9—13.6)	9.7 (8.1—14.9)
Platelets, 1,000/mm3*	246 (200—305)	258 (215—305)	140 (102—201)	161 (144—218)
Sodium, mEq/L*	139.0 (138.0—141.0)	140.0 (138.0—141.0)	140.0 (139.0—142.0)	145.0 (140.0—147.0)
BUN, mg/dL*	16 (13—20)	14 (11—18)	16 (13—25)	23 (15—42)
Creatinine, mg/dL*	0.80 (0.65—0.94)	0.68 (0.59—0.86)	0.56 (0.40—0.80)	0.65 (0.39—0.79)
ALT, U/L*	17 (12—24)	14 (11—21)	19 (11—30)	18 (13—38)
AST, U/L*	21 (17—28)	19 (16—26)	23 (18—35)	22 (21—37)
Albumin, g/dL*	4.00 (3.70—4.30)	4.00 (3.70—4.30)	3.35 (3.10—3.90)	4.05 (3.60—4.30)
Total bilirubin, mg/dL*	0.50 (0.30—0.70)	0.40 (0.30—0.50)	0.85 (0.50—1.50)	0.80 (0.60—1.00)
INR	1.00 (1.00—1.10)	1.00 (1.00—1.10)	1.20 (1.10—1.20)	1.20 (1.10—1.30)
Donor				
Age, years	33 (23—44)	38 (31—46)	36.5 (25.0-43.3)	34.5 (25.8-44.3)
Female	90 (28.8%)	20 (43.5%)	18 (47.4%)	5 (35.7%)
Cause of death				
Anoxia	128 (40.9%)	23 (50.0%)	13 (34.2%)	7 (50.0%)
head trauma	108 (34.5%)	13 (28.3%)	16 (42.1%)	2 (14.3%)
Stroke	49 (15.7%)	6 (13.0%)	8 (21.1%)	4 (28.6%)
Other	28 (8.9%)	4 (8.7%)	1 (2.6%)	1 (7.1%)

Continuous data are shown as median (range) and discrete data are shown as number (%). BMI, body mass index; BSA, body surface area; CKD, chronic kidney disease; PRA, panel reactive antibody; ILD; interstitial lung disease; COPD, chronic obstructive pulmonary disease; PAH, pulmonary arterial hypertension; ARDS, Acute Respiratory Distress Syndrome; WBC, white blood cell; BUN, blood urea nitrogen; AST, aspartate aminotransferase; ALT, Alanine aminotransferase; INR, international normalized ratio.
*Unknown cases were excluded

Supplemental Table 2. Intraoperative outcomes of lung transplant recipients

Variable	No VV-ECMO bridge		VV-ECMO bridge	
	cPRA negative (n=313)	cPRA positive (n=46)	cPRA negative (n=38)	cPRA positive (n=14)
Intraoperative outcome				
Operative time (hours)*	5.5 (4.3—7.1)	5.6 (4.4—7.3)	8.2 (5.4—9.5)	7.6 (6.8—10.0)
Intra-op blood transfusion(unit)				
pRBC	0.0 (0.0—2.0)	1.0 (0.0—2.0)	7.0 (5.0—10.0)	11.0 (7.0—16.0)
FFP	0.0 (0.0—0.0)	0.0 (0.0—0.0)	2.0 (1.0—6.0)	3.5 (2.0—6.0)
Plt	0.0 (0.0—0.0)	0.0 (0.0—0.0)	2.0 (1.0—4.0)	2.0 (1.0—4.0)
Ischemic time (hours)*	5.1 (4.1—6.1)	5.0 (3.8—5.9)	5.8 (5.2—6.5)	6.0 (5.8—6.6)
VA-ECMO use	183 (58.5%)	28 (60.9%)	35 (92.1%)	14 (100.0%)
Postoperative outcomes				
de novo DSA*	38 (12.2%)	20 (43.5%)	7 (18.4%)	5 (35.7%)
PGD				
Any grade	170 (54.3%)	25 (54.3%)	28 (73.7%)	12 (85.7%)
Grade3	32 (10.2%)	2 (4.3%)	19 (50.0%)	5 (35.7%)
AKI	142 (45.4%)	16 (34.8%)	26 (68.4%)	9 (64.3%)
Dialysis	37 (11.8%)	8 (17.4%)	13 (34.2%)	3 (21.4%)
CVA	11 (3.5%)	0 (0.0%)	1 (2.6%)	0 (0.0%)
Bowel ischemia	4 (1.3%)	1 (2.2%)	1 (2.6%)	0 (0.0%)
Digital ischemia	4 (1.3%)	0 (0.0%)	4 (10.5%)	1 (7.1%)
DVT	166 (53.0%)	21 (45.7%)	21 (55.3%)	10 (71.4%)
PE	40 (12.8%)	10 (21.7%)	7 (18.4%)	1 (7.1%)
ICU stay (days)*	6.0 (4.0—12.0)	9.0 (6.0—14.0)	19.5 (12.0—25.0)	19.5 (12.0—26.0)
Post transplant ventilator (days)*	2.0 (1.0—3.0)	3.0 (2.0—5.0)	4.0 (2.0—15.0)	6.5 (2.0—17.0)
Hospital stay (days)	16.0 (11.0—29.0)	16.0 (12.0—31.0)	37.5 (24.0—48.0)	35.0 (24.0—40.0)
Follow-up period (days)	506.0 (213.0—1,020.0)	698.0 (304.0—932.0)	632.5 (312.0—1,381.0)	787.0 (411.0—1,135.0)

Continuous data are shown as median (interquartile range) and discrete data are shown as number (%). pRBC, packed red blood cells; FFP, fresh frozen plasma; Plt, platelets; VA ECMO, veno-arterial extracorporeal membrane oxygenation; DSA, donor specific antibody; PGD, primary graft dysfunction; AKI, acute kidney injury; CVA, cerebrovascular attack; DVT, deep vein thrombosis; PE, pulmonary embolism; ICU, intensive care unit. *Unknown cases were excluded

Supplemental Table3. Univariate and multivariate logistic regression analysis as a predictor of PGD grade3

Variable	Univariate			Multivariate		
	Odds ratio	95% CI	p value	Odds ratio	95% CI	p value
Recipient factors						
Age, years	0.97	0.9–1.0	0.001	0.99	1.0–1.0	0.31
Female	1.28	0.7–2.2	0.39			
BMI, kg/m2	1.01	1.0–1.1	0.64			
BSA, m2	1.25	0.4–3.8	0.70			
Smoking history	0.76	0.4–1.3	0.34			
Hypertension	1.03	0.6–1.8	0.91			
Diabetes	0.95	0.5–1.8	0.88			
CKD	1.80	0.7–4.4	0.19			
Pre-op blood transfusion						
within 4weeks	1.22	1.1–1.4	0.0003			
within 1week	2.21	1.5–3.2	<.0001	1.89	1.3–2.7	0.001
cPRA	0.78	0.3–1.8	0.56			
Etiology						
ILD	1.00	0.6–1.8	0.99			
ARDS	2.99	1.5–6.0	0.002	1.20	0.5–3.0	0.69
Laboratory						
Hemoglobin, g/dL*	0.84	0.7–0.9	0.004			
WBC, 1,000/mm3*	1.03	1.0–1.1	0.36			
Platelets, 1,000/mm3*	1.00	1.0–1.0	0.004			
Sodium, mEq/L*	1.07	1.0–1.2	0.09			
BUN, mg/dL*	1.03	1.0–1.1	0.05			
Creatinine, mg/dL*	1.73	0.6–4.9	0.30			
ALT, U/L*	1.01	1.0–1.0	0.27			
AST, U/L*	1.02	1.0–1.0	0.01			
Albumin, g/dL*	0.37	0.2–0.6	0.0002	0.47	0.3–0.8	0.01
Total bilirubin, mg/dL*	2.07	1.3–3.2	0.001			
INR	3.27	1.0–10.9	0.05			
Donor						
Age, years	1.01	1.0–1.0	0.40			
Female	1.72	1.0–3.0	0.06			
Intraoperative outcome						
Operative time (hours)*	1.21	1.1–1.4	0.01	1.01	0.8–1.2	0.93
Ischemic time (hours)*	1.02	0.9–1.1	0.63			

DSA, donor specific antibody; BMI, body mass index; BSA, body surface area; CKD, chronic kidney disease; PRA, Panel Reactive Antibody; WBC, white blood cell; BUN, blood urea nitrogen; AST, aspartate aminotransferase; ALT, Alanine aminotransferase; INR, international normalized ratio. *Unknown cases were excluded

Supplemental Table4. Univariate and multivariate cox proportional hazard

Variable	Univariate analysis			Multivariate analysis		
	Hazard Ratio	95% CI	p value	Hazard Ratio	95% CI	p value
Recipient factors						
Age, years	1.01	0.99–1.03	0.17			
Female	1.08	0.72–1.61	0.72			
BMI, kg/m2	1.03	0.99–1.08	0.15			
BSA, m2	1.06	0.45–2.50	0.89			
Smoking history	1.11	0.74–1.65	0.62			
Hypertension	1.04	0.70–1.56	0.84			
Diabetes	1.42	0.94–2.14	0.10			
CKD	2.04	1.13–3.68	0.02	1.34	0.73–2.46	0.34
Bilateral	0.56	1.20–2.68	0.004	0.51	0.34-0.77	0.001
cPRA	0.95	0.54–1.67	0.85			
Etiology						
ILD	1.14	0.76–1.72	0.52			
ARDS	0.70	0.37–1.31	0.26			
Laboratory						
Hemoglobin, g/dL*	1.00	0.92–1.08	0.98			
WBC, 1,000/mm3*	0.97	0.92–1.03	0.31			
Platelets, 1,000/mm3*	1.00	1.00–1.00	0.80			
Sodium, mEq/L*	1.02	0.96–1.08	0.56			
BUN, mg/dL*	1.00	0.98–1.03	0.74			
Creatinine, mg/dL*	2.77	1.26–6.10	0.01			
ALT, U/L*	1.01	1.00–1.02	0.22			
AST, U/L*	1.01	1.00–1.01	0.07			
Albumin, g/dL*	0.71	0.49–1.03	0.07			
Total bilirubin, mg/dL*	1.16	0.87–1.55	0.30			
INR	1.18	0.42–3.34	0.76			
Donor						
Age, years	1.01	1.00–1.03	0.14			
Female	1.16	0.76–1.76	0.50			
Intraoperative outcome						
Operative time (hours)*	0.97	0.88–1.08	0.58			
Ischemic time (hours)*	0.91	0.80–1.03	0.15			
Postoperative outcomes						
PGD						
Any grade	1.38	0.92–2.08	0.12			
Grade3	2.99	1.90–4.70	<.0001	2.88	1.80–4.59	<.0001
AKI	2.20	1.46–3.31	0.0002			
Dialysis	2.43	1.26–4.68	0.01			
DVT	2.27	1.44–3.59	0.0004	1.98	1.24–3.15	0.004
PE	1.26	0.75–2.13	0.39			

BMI, body mass index; BSA, body surface area; CKD, chronic kidney disease; PRA, Panel Reactive Antibody; WBC, white blood cell; BUN, blood urea nitrogen; AST, aspartate aminotransferase; ALT, Alanine aminotransferase; INR, international normalized ratio; DSA, donor specific antibody; PGD, primary graft dysfunction; AKI, acute kidney injury; DVT, deep vein thrombosis; PE, pulmonary embolism; ICU, intensive care unit. *Unknown cases were excluded. *Unknown cases were excluded
